# Supplementary material for: Comparative Transcriptome Analysis Reveals Complex Physiological Response and Gene Regulation in Peanut Roots and Leaves under Manganese Toxicity Stress
Source: Int J Mol Sci. 2023 Jan 6;24(2):1161. doi: 10.3390/ijms24021161 (PMC9867376; doi:10.3390/ijms24021161)
Supplement: Supplementary file 1 [file ijms-24-01161-s001.zip › Figures S1 and S2.pdf]

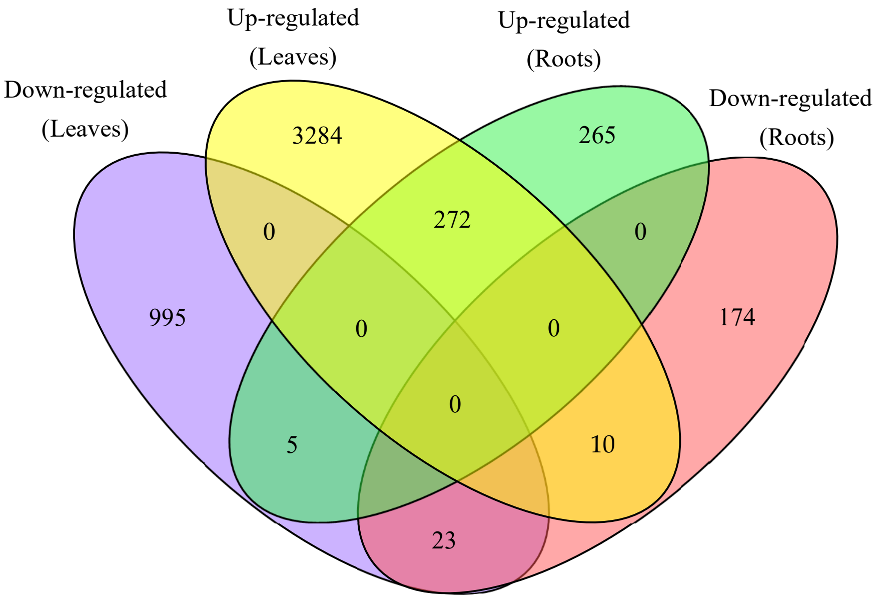

Figure S1. Analysis using a Venn diagram of the genes that differ in expression between Mn toxicity and control treatments in the peanut leaves and roots.

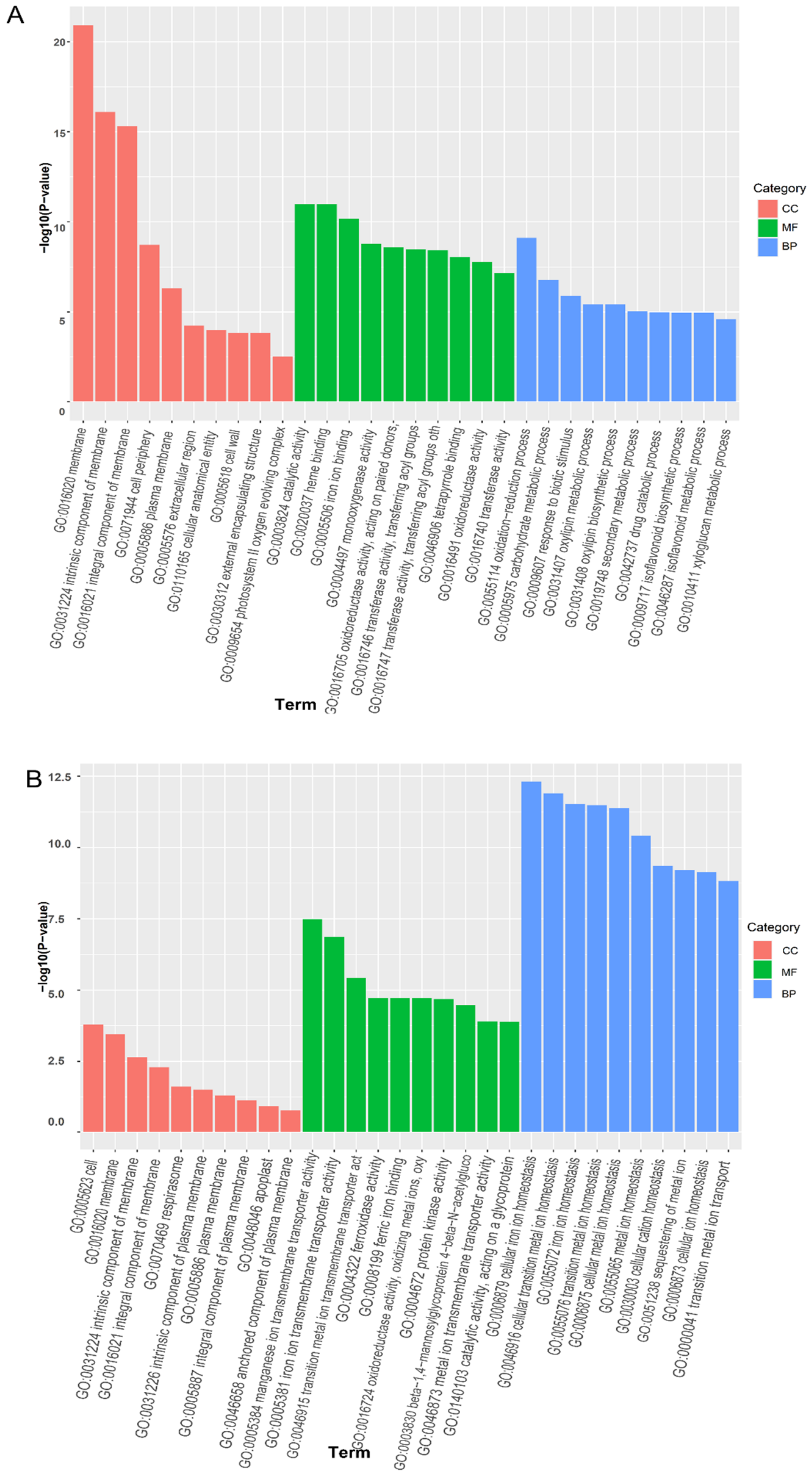

Figure S2. Histogram of GO enrichment analysis. A: CK leaves and 300 μM Mn leaves, B: CK roots and 300 μM Mn roots. The horizontal coordinate was the term of Go level2 level and the vertical coordinate was the -log10 (p-value) of enrichment for each term.
